# Ultrasound-assisted formation of composite materials from fish scale waste hydroxyapatite in the presence of gamma-irradiated chitosan for the removal of malachite green†

**DOI:** 10.1039/d4ra03102d

**Published:** 2024-10-01

**Authors:** Nattaporn Keanjun, Thitirat Rattanawongwiboon, Phitchan Sricharoen, Sakchai Laksee, Naengnoi Saengsane, Yanisa Thepchuay, Paweena Porrawatkul, Rungnapa Pimsen, Arnannit Kuyyogsuy, Prawit Nuengmatcha, Saksit Chanthai, Maliwan Subsadsana, Nunticha Limchoowong

**Affiliations:** a Department of Chemistry, Faculty of Science, Srinakharinwirot University Bangkok 10110 Thailand nunticha@g.swu.ac.th; b Nuclear Technology Research and Development Center, Thailand Institute of Nuclear Technology (Public Organization) Nakhon Nayok 26120 Thailand; c Division of Health, Cosmetic and Anti-Aging Technology, Faculty of Science and Technology, Rajamangala University of Technology Phra Nakhon Bangkok 10800 Thailand phitchan.s@rmutp.ac.th; d Nanomaterials Chemistry Research Unit, Department of Chemistry, Faculty of Science and Technology, Nakhon Si Thammarat Rajabhat University Nakhon Si Thammarat 80280 Thailand; e Materials Chemistry Research Center, Department of Chemistry and Center of Excellence for Innovation in Chemistry, Faculty of Science, Khon Kaen University Khon Kaen 40002 Thailand; f Program of Chemistry, Faculty of Science and Technology, Nakhon Ratchasima Rajabhat University Nakhon Ratchasima 30000 Thailand

## Abstract

The fish processing sector produces millions of tons of trash annually—a biologically dangerous substance that could eventually turn into a source of pathogenic contamination. This work successfully shows how to extract tilapia fish scale hydroxyapatite with ultrasonic assistance and modify it using gamma-irradiated chitosan to remove malachite green from water samples. The prepared adsorbent was characterized by Fourier transform infrared spectroscopy, X-ray diffraction, X-ray fluorescence, scanning electron microscopy, energy dispersive spectroscopy, transmission electron microscopy, thermogravimetric analysis and dynamic light scattering. Isotherm modeling was employed to investigate the sorption process of malachite green. The results revealed that the adsorbent could be used to remove malachite green in aqueous media, with a maximum adsorption capacity of 285.7 mg g^−1^. A pseudo-second-order model was then fitted to the kinetic data. The *R*^2^ value of 0.9851 obtained indicated that the adsorption behavior was consistent with the Langmuir model. Analysis of the computed thermodynamic parameters revealed that the adsorption of the dye was a spontaneous and exothermic process. Proper waste management practices not only ensure environmental responsibility but also contribute to positive community relations by minimizing the impact on the local environment.

## Abstract 2

The fish processing sector produces millions of tons of trash annually—a biologically dangerous substance that could eventually turn into a source of pathogenic contamination.

## Graphical abstract

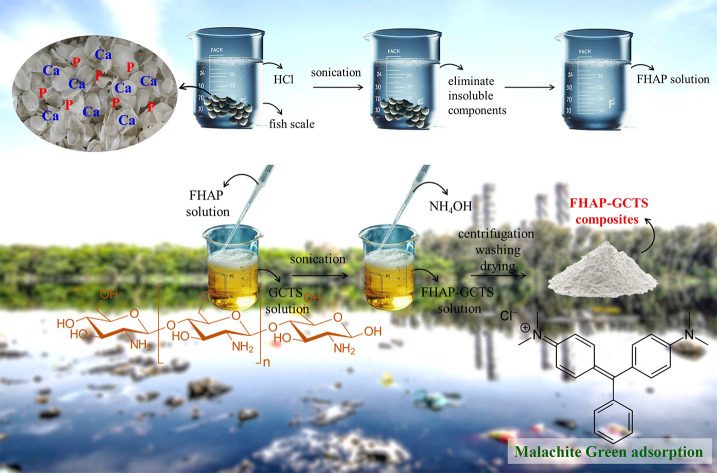

## 1. Introduction

Tilapia's prominence as a farmed fish species is assured, with projections indicating even greater production in the coming years. The global tilapia market's value is expected to rise by 3–5%.^1^ Humans consume a large quantity of fish, directly leading to the generation of fish waste, which, if not managed properly, can result in environmental issues and waste concerns. There are several aspects to consider regarding the potential issues associated with fish scale residues, such as the following: (1) organic waste, including scales, may decompose and release nutrients into the environment, impacting water quality if discharged into water bodies, and (2) the accumulation of fish scale residues can produce unpleasant odors, attracting scavengers and causing aesthetic issues in the surrounding area. This can be a concern for both local communities and the facilities responsible for waste disposal.

Adequate waste management of fish scale residues involves proper disposal, recycling, or alternative uses. Research and innovation focus on sustainable solutions, including technologies to extract valuable components and finding new applications for the residues. Hydroxyapatite derived from Nile tilapia fish scales (FHAP, Ca_10_(PO_4_)_6_(OH)_2_) was identified as ecological and biocompatible material that has a crystalline structure composed of calcium and phosphate ions, and it closely resembles the mineral component of natural bone.^2–4^ It has gained attention for use in various applications, particularly in the fields of biomaterials, medicine, and environmental science. FHAP can potentially offer a cost-effective alternative to synthetic hydroxyapatite produced through chemical methods.^5^ The abundance of fish scales from tilapia processing makes it a readily available and economically viable source. The contribution of hydroxyapatite to the adsorption process was elucidated. Scales from various fish species were identified as having been utilized as adsorbents for heavy metals, radionuclides and dyes removal.^6^ Hydroxyapatite has been recommended in numerous studies as a suitable adsorbent for the removal of heavy metal ions, including Cd^2+^, Co^2+^, Cr^6+^, Cu^2+^, Fe^3+^, Hg^2+^, Ni^2+^, Pb^2+^, Zn^2+^ and As^5+^, from aqueous solutions.^7^ The maximum adsorption capacity of hydroxyapatite-based adsorbents can be enhanced through surface modification. For instance, calcined magnetic layered double hydroxide/hydroxyapatite, synthesized *via* the co-precipitation method, has been utilized for the adsorption of U(vi) radionuclides, exhibiting a maximum adsorption capacity of 208 mg g^−1^.^8^ Additionally, spherical hydroxyapatite/polyvinyl butyral beads have been reported to possess a maximum adsorption capacity of 11.35 mg g^−1^ for Co(ii) radionuclides.^9^ A modified form of chitosan, a naturally occurring polymer derived from chitin and found in the exoskeletons of crustaceans such as crabs, shrimp, and other arthropods, was designated as gamma-irradiated chitosan (GCTS).^10,11^ Chitosan itself is created by deacetylating chitin, a process that removes the acetyl groups from chitin molecules. GCTS is produced by subjecting chitosan to gamma irradiation. One type of ionizing radiation that can change a material's chemical and physical characteristics is gamma irradiation. In the case of chitosan, its molecular structure and properties were altered by gamma irradiation, resulting in a material with improved properties compared to untreated chitosan. It was observed that gamma-irradiated chitosan exhibited greater solubility in an aqueous configuration than non-irradiated chitosan.^12,13^ It was also found in the study that a significant reduction in the molecular weight of chitosan occurred as the gamma radiation dose was increased. Specifically, doses ranging from 5 to 50 kGy resulted in a proportional decrease in molecular weight due to chain scission.^14^ Additionally, it was shown that solubility of chitosan was increased by breaking down its molecular structure, with doses up to 100 kGy.^15^ These changes can be beneficial for various applications, including biomedical and industrial uses, where specific solubility and molecular weight profiles are required. The motivation for utilizing a chitosan composite with hydroxyapatite derived from fish waste scales to eliminate malachite green was driven by several key factors. Firstly, the environmental and health concerns associated with malachite green prompted stringent regulations on its usage, necessitating the development of wastewater remediation methods. Secondly, fish waste scales, often discarded as a by-product of the food restaurants, were identified as a potential resource. By innovatively re-purposing these scales to extract hydroxyapatite, waste could be reduced. Additionally, the excellent adsorption properties and biocompatibility of chitosan suggested that its combination with hydroxyapatite could enhance the capacity for malachite green adsorption while utilizing alternative materials.

The objective of the present study is to illustrate that hydroxyapatite–chitosan composites (FHAP–GCTS) can be synthesized by employing an ultrasonic technique on a hydroxyapatite-based solution derived from fish scales, combined with gamma chitosan (chitosan exposed to gamma radiation at sterilizing dosages of 40 kGy), and initiated with a base solution. Fourier transform infrared spectroscopy, X-ray diffraction, X-ray fluorescence, scanning electron microscopy, energy dispersive spectroscopy, transmission electron microscopy, thermogravimetric analysis and dynamic light scattering were used to characterize the FHAP–GCTS before the spectroscopic measurement of malachite green adsorption. After that, the adsorption optimization, isotherm, kinetics and thermodynamics were all examined.

## 2. Experimental

### 2.1. Materials

The subsequent resources were used exactly as supplied: tilapia fish scales obtained from a local market, ammonium hydroxide (28%, Aldrich), hydrochloric acid (37%, Aldrich), malachite green (Aldrich), and acetic acid (99.6%, Merck). Chitosan exposed to gamma radiation at sterilizing dosages of 40 kGy, Mw = 190 kDa and DD = 95%, the Thailand Institute of Nuclear Technology (Public Organization) provided it. Throughout the experiment, deionized water (DI) with a resistivity of 18.2 MΩ cm and a conductivity of approximately 0.055 μS cm^−1^ was utilized.

### 2.2. Apparatus

Using an IR spectrometer (spectra Two, PerkinElmer Scientific, USA) was acquired. A Bruker AXS D8 ADVANCE X-ray diffractometer (Bruker Optics, Germany) was used to scan 2*θ* in the 10°–50° range. Monochromatic Cu Kα radiation (*λ* = 0.15406 nm) was used to acquire XRD patterns. Surface morphology and EDS spectrum were analyzed using a field emission scanning electron microscope (FE-SEM, HITACHI SU5000, Japan) and EDS (Oxford Instruments, United Kingdom). TEM images were obtained with a Tecnai G2 20 operating at 200 kV (FEI, USA). The thermal decomposition behavior was studied employing a Mettler Toledo thermogravimetric analyzer (TGA/DSC2/LF/1100, Switzerland). Samples weighing about 5 mg were heated in aluminum oxide pans at a rate of 20 °C per minute while nitrogen gas flow was maintained at 90 mL min^−1^. X-ray fluorescence (XRF) analysis was conducted using a Micro-XRF Spectrometer (Bruker M4 Tornado, Germany). Synthesis and adsorption processes were conducted in an ultrasonic bath (Cavitator Ultrasonic Cleaner Model 5.5S, Mettler Electronics, USA) operating at a frequency of 35 kHz. All absorbance measurements were carried out utilizing a UV–Visible spectrophotometer (UV5100, Metash, China) with a 10 mm optical path length employing a 3.5 mL quartz cuvette (Fisher Scientific, USA).

### 2.3. Hydroxyapatite-based solution derived from tilapia fish scales

**Scheme 1:**
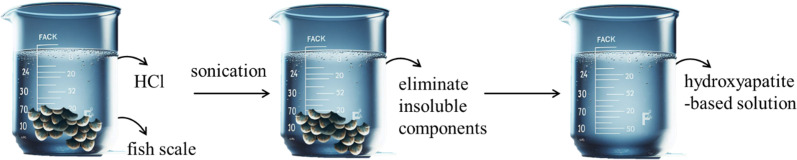
Preparation of the FHAP solution (fish scale hydroxyapatite-based solution).

The tilapia fish scales had been dried after being cleaned with deionized water. Subsequently, 2.5 g of the dried scales were added to 100 mL of 0.8 M hydrochloric acid as the extraction solvent in a glass beaker to extract hydroxyapatite sources, such as Ca (calcium) and P (phosphorus) elements. Sonication, lasting 30 minutes, was utilized as a tool to accelerate the extraction process, after which the mixture was filtered using filter paper to remove insoluble components. A clear solution rich in Ca and P elements was obtained, referred to as the FHAP solution (fish scale hydroxyapatite-based solution) (Scheme 1).

### 2.4. Preparation of fish scale hydroxyapatite–gamma chitosan composites (FHAP–GCTS)

**Scheme 2:**
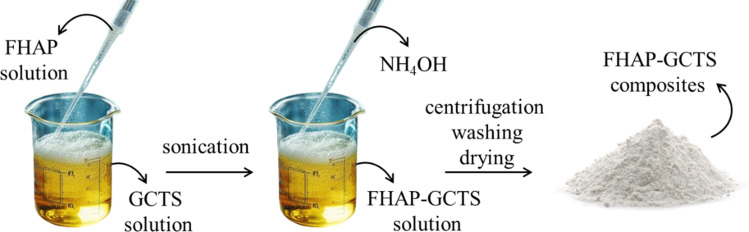
Preparation of fish scale hydroxyapatite–gamma chitosan composites (FHAP–GCTS).

A 2.5 g sample of GCTS powder had been dissolved in 100 mL of a 2% acetic acid (CH_3_COOH) solution in deionized water. The mixture was then stirred to homogenize until a clear solution was formed. Following this, an FHAP solution from Section 2.3 was added to the GCTS solution, and the resulting blend underwent sonication for 30 minutes to create a uniform suspension. Subsequently, 28% ammonium hydroxide (NH_4_OH) was introduced dropwise into the solution to initiate the formation of precipitated composites. The pH was adjusted to 12, and sonication was continued for an additional 60 minutes to complete the dispersed synthesis. The prepared composites were subjected to centrifugation to separate the solid composites, which were then washed multiple times with deionized water and subsequently baked at 70 °C to dry. The resultant solid sample was identified as an FHAP–GCTS composite (Scheme 2).

### 2.5. Adsorption test

Ultrasonic techniques offer valuable tools for investigating and improving adsorption processes, providing insights into adsorption properties of adsorbent materials.^16,17^ In the adsorption test, 0.02 g of dried FHAP–GCTS powder was introduced into 10 mL of a 200 mg L^−1^ MG, followed by sonication for 10 minutes. Subsequently, the composite powders were separated. The MG concentrations were assessed using a UV-Vis spectrometer at 618 nm. The adsorption capacity was optimized under various parameters such as pH, FHAP–GCTS dosage, time, initial adsorbate concentration and temperature. The adsorption capacity at equilibrium (*q*_e_, mg g^−1^) was determined using eqn (1):^18^1
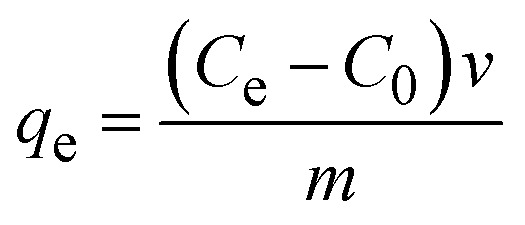
where *C*_0_ and *C*_e_ are the initial concentration and the final concentration in the solution at equilibrium of the adsorbate (mg L^−1^), respectively; *V* (L) and *m* (g) are the solution volume and the absorbent mass.

The adsorption equilibrium properties were examined using the Freundlich (eqn (3)) and Langmuir (eqn (2)) isotherms:^18^2
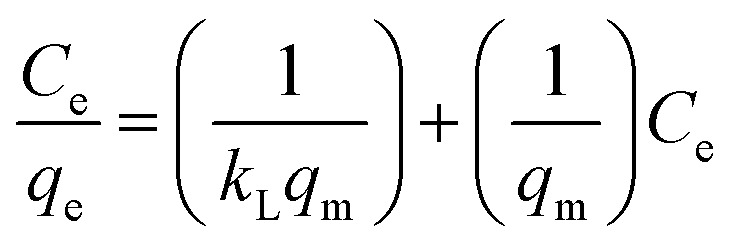
3
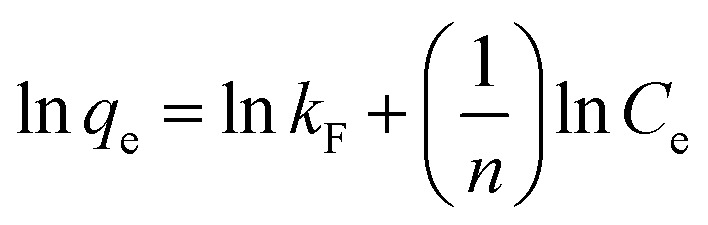
where *q*_e_ (mg g^−1^), *q*_m_ (mg g^−1^), and *C*_e_ (mg L^−1^) are the adsorption capacity, maximum adsorption capacity, and absorbate concentration at equilibrium, respectively; *K*_L_ (L mg^−1^) and *K*_F_ (mg g^−1^) are the Langmuir adsorption and Freundlich constants, respectively; 1/*n* is the Freundlich constant attributed to the rough surface of the pollutant.

Pseudo-first-order (eqn (4)) and pseudo-second-order (eqn (5)), the two primary forms of adsorption kinetic models,^18,19^ were taken into consideration to study the adsorption process:

4 ln(*C*_e_ − *Q*_e_) = ln *C*_e_ − *k*_1_*t*

5
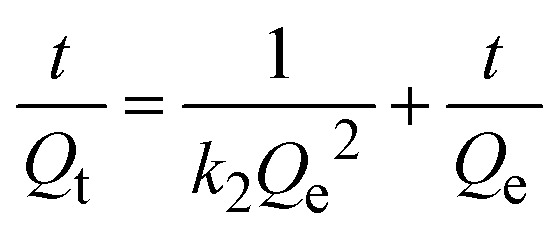
where *C*_e_ is the equilibrium concentration, *t* is time, *k*_1_ and *k*_2_ are the rate constants of the pseudo-first-order and pseudo-second-order adsorption (min^−1^), respectively. *Q*_*t*_ is the amount of adsorbate on the adsorbent at time *t* (mg g^−1^). *Q*_e_ is the amount of adsorbate on the adsorbent at equilibrium (mg g^−1^).

The study focused on the adsorption thermodynamic parameters, including enthalpy change (Δ*H*°), entropy change (Δ*S*°), and Gibbs free energy change (Δ*G*°). The following formulas were used to calculate these parameters:^20^

6 Δ*G*° = −*RT* ln *K*_eq_

7 Δ*G*° = Δ*H*° − *T*Δ*S*°

8 ln *K*_eq_ = Δ*S*°/*R* − Δ*H*°/*RT*

where *T* is absolute temperature and *R* is the gas constant. The corresponding values of Δ*G*° can be determined at different experimental temperatures.

## 3. Results and discussion

### 3.1. Fourier transform infrared spectroscopy (FTIR)

**Fig. 1:**
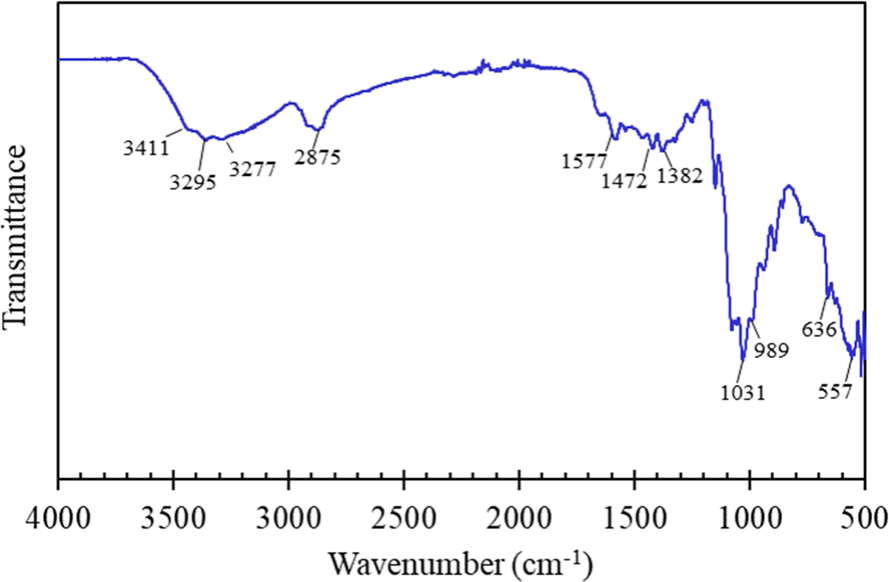
FTIR spectrum of FHAP–GCTS composites.

Fig. 1 illustrated the chemical structural characteristics of the FHAP–GCTS composites. Analysis revealed that the composites retained all the characteristic peaks associated with both chitosan and hydroxyapatite. The chitosan portion displayed bands at 3411 and 3295 cm^−1^ (O–H and N–H stretching), 2875 cm^−1^ (C–H stretching), 1577 cm^−1^ (N–H stretching), and 1472 and 1382 cm^−1^ (C=O and C–O stretching). The hydroxyapatite portion of the spectrum exhibited bands characteristic of water adsorption at 3277 cm^−1^ and PO_4_^3−^ groups at 1031 cm^−1^ (asymmetrical P–O stretching), 989 cm^−1^ (symmetrical P–O stretching), and 636 and 557 cm^−1^ (O–P–O bending).^12–24^ The broadening observed around 1031 cm^−1^ suggested the presence of the GCTS polymer and its interaction with PO_4_^3−^ groups of FHAP.^25^ This, therefore, indicated the likelihood of physical interactions, particularly electronic forces and hydrogen bonds, being established between FHAP and GCTS during the composite formation.

### 3.2. X-ray diffraction (XRD)

As depicted in ESI Fig. S1,† the XRD pattern of the FHAP–GCTS composite reveals the distinct crystallographic phases of both components, along with evidence of an amorphous structure. The presence of weak diffraction peaks at 19.68° and 26.09° corresponds to the (110) and (220) planes of chitosan, indicating its integration into the composite with a relatively low degree of crystallinity. Additionally, the broad hump in the XRD baseline suggests the presence of an amorphous phase, which may arise from the disordered regions of chitosan or poorly crystalline hydroxyapatite. In contrast, the sharper and more prominent peaks observed at 32.31°, 37.17°, 40.09°, 47.25°, and 49.54° confirm the primary crystalline structure of hydroxyapatite, derived from fish scales, and align well with the standard JCPDS 00-009-0432 pattern for hydroxyapatite.^26^ These peaks correspond to the (211), (301), (310), (222), and (213) planes of hydroxyapatite in the composite, indicating that hydroxyapatite is the dominant phase, while the presence of amorphous material contributes to the overall composite structure.

### 3.3. X-ray fluorescence (XRF)

**Fig. 2:**
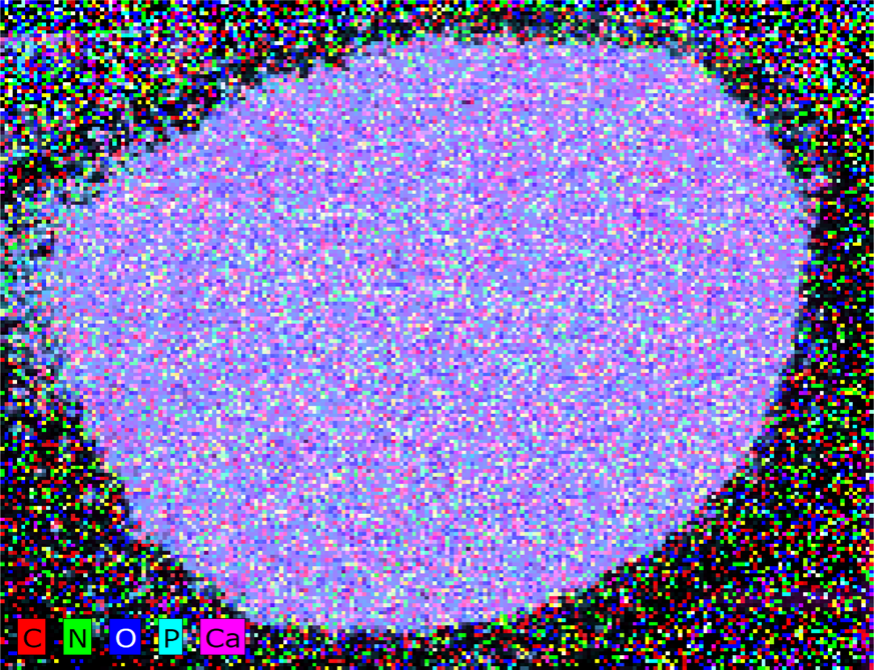
XRF mapping of FHAP–GCTS composites.

When applied to FHAP–CTS composites, XRF can provide information about the elemental composition of the material.^27^ This is illustrated in Fig. 2. The elemental analysis of FHAP–CTS revealed the presence of oxygen (O), phosphorus (P), and calcium (Ca) as characteristic elements in hydroxyapatite, along with carbon (C) and nitrogen (N), which are typical of chitosan. The mapping results confirm the uniform distribution of hydroxyapatite and chitosan, covering the entire surface of the FHAP–CTS composite.

### 3.4. Field emission scanning electron microscope (SEM) and energy dispersive spectroscopy (EDS)

**Fig. 3:**
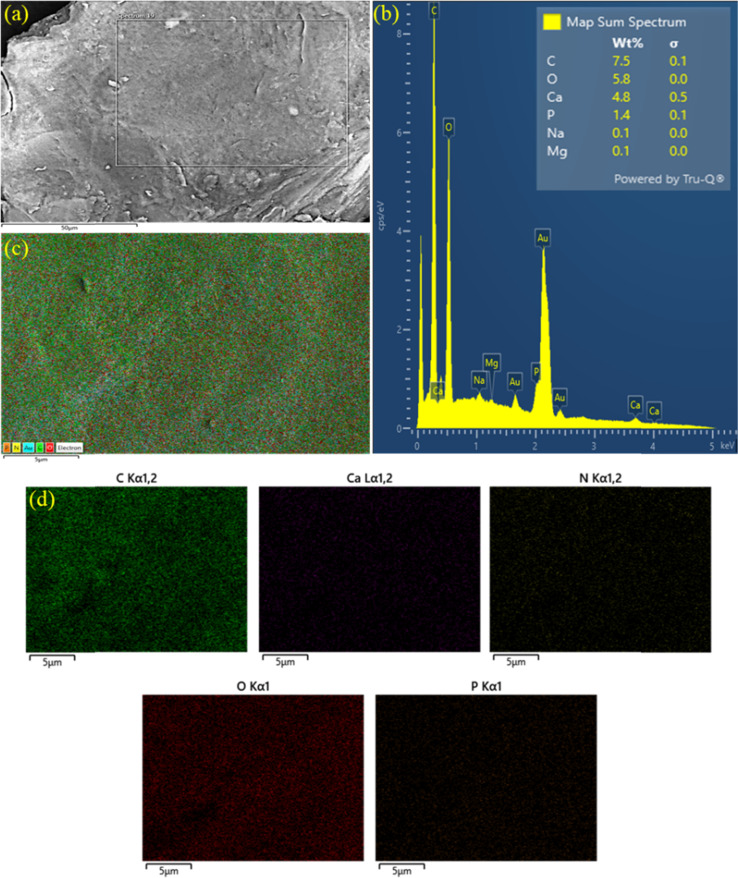
FE-SEM (a), EDS spectrum (b) and EDS mapping (c and d) of FHAP–GCTS.

The FE-SEM images (Fig. 3a) highlight the smooth surfaces of the studied composites. The examination of the chemical composition of the FHAP–GCTS composites was carried out through the utilization of EDS investigations.^28^ In the EDS spectrum (Fig. 3b), the presence of elements such as carbon (C, 7.5), oxygen (O, 5.8), calcium (Ca, 4.8), and phosphorus (P, 1.4) was observed mainly at concentrated atomic percentages. The occurrence of peaks corresponding to C and O in GCTS validates the successful modification of FHAP with Ca and P. Consequently, the modified composite holds potential for dye removal from samples. The outcomes of the EDS studies, along with the elemental distribution maps of the principal elements, are presented. The analysis results were indicative of the presence of carbon, oxygen, calcium, phosphorus, and nitrogen within the composites. These elements are all characteristic of both FHAP and GCTS. Furthermore, the uniform distribution of these chemical elements across the composites was affirmed by the elemental distribution maps featured in Fig. 3c and d.

### 3.5. Transmission electron microscopy (TEM)

**Fig. 4:**
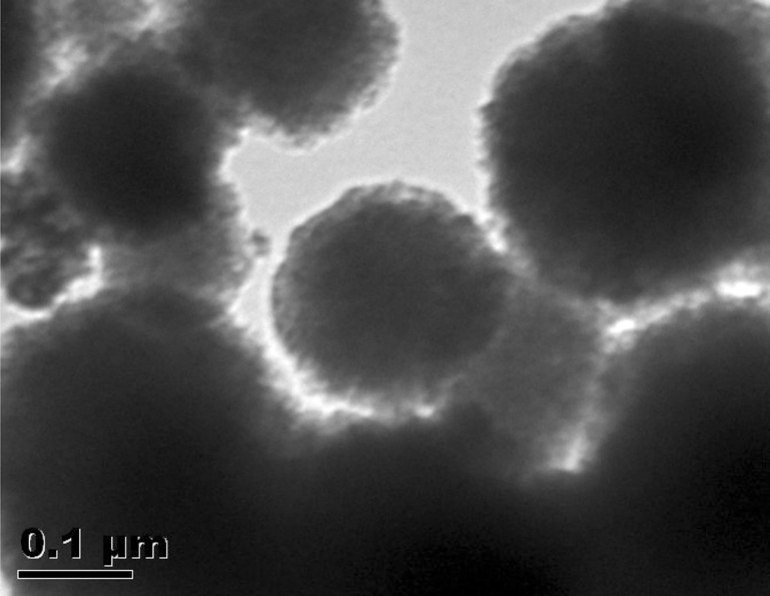
TEM micrographs of FHAP–GCTS.

Fig. 4 shows the TEM images of FHAP–GCTS, showcasing a spherical shape with fluctuating sizes ranging from 0.15 to 3 μm. The fluctuating sizes and morphologies observed in the hydroxyapatite–chitosan composites can be attributed to several factors inherent to the synthesis process and the resulting composite material itself. The resulting material's morphology may deviate from those seen in earlier studies in which hydroxyapatite displayed needle-shaped,^29^ rod-shaped,^30^ rice-shaped, and round-shaped structures.^31^ These variations in morphology in comparison to this study could be attributed to the chosen one-pot ultrasonic synthesis method, which might include the use of a hydroxyapatite-based solution combined with a chitosan solution, followed by co-assembly and subsequent co-precipitation.

### 3.6. Thermogravimetric analysis (TGA)

**Fig. 5:**
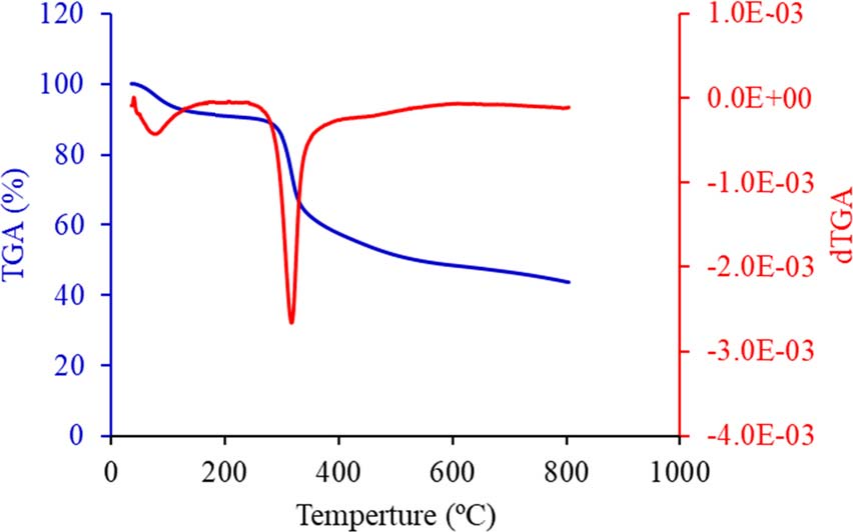
TGA and dTGA curve of FHAP–GCTS.

Thermogravimetric analysis (TGA) was employed to elucidate the thermal degradation behavior and identify the organic functional groups associated with the FHAP. Fig. 5 presents the TGA curve (blue line) obtained for the FHAP–GCTS composites under an air atmosphere at a temperature range of 50 to 800 °C. The first stage was identified as the dehydration of physically held water, which started at a comparatively low temperature in the range of 50–100 °C. Generally, the main chain's thermal breakdown and degradation, as well as the chitosan molecules' deacetylation, are responsible for the predominant mass loss in pure chitosan cases, which is measured between 228 and 400 °C.^32^ Compared to pure chitosan, this mass loss stage happens at a lower temperature in the case of FHAP–GCTS composites. This lower temperature phenomenon could be explained by the inorganic FHAP particles on the surface facilitating better heat transfer to the GCTS matrix, which in turn accelerates the decomposition of organic chitosan. Owing to the breakdown of broken pieces and gradual char oxidation, FHAP–GCTS displayed an additional mass loss stage in the 280–600 °C range. Lastly, the dehydroxylation of FHAP during its thermal breakdown may be linked to the remaining mass loss at temperatures higher than 600 °C. To determine which transitions in the dTGA curve (red line) corresponded to specific thermal events in hydroxyapatite and its composites with chitosan, the temperature ranges typically associated with these processes were examined. The initial peak around 100–200 °C was associated with the drying of the sample, including the release of loosely bound and crystal-bound water. The second peak, occurring between 200–500 °C, indicated the release of structural water from hydroxyapatite and its conversion to apatite or other phases like oxyhydroxyapatite or tricalcium phosphate (TCP). By identifying these transitions, an understanding of the thermal behavior of the hydroxyapatite-chitosan composite and the temperatures at which significant changes occurred was achieved.

### 3.7. Adsorption studies

#### 3.7.1. Effect of pH

**Fig. 6:**
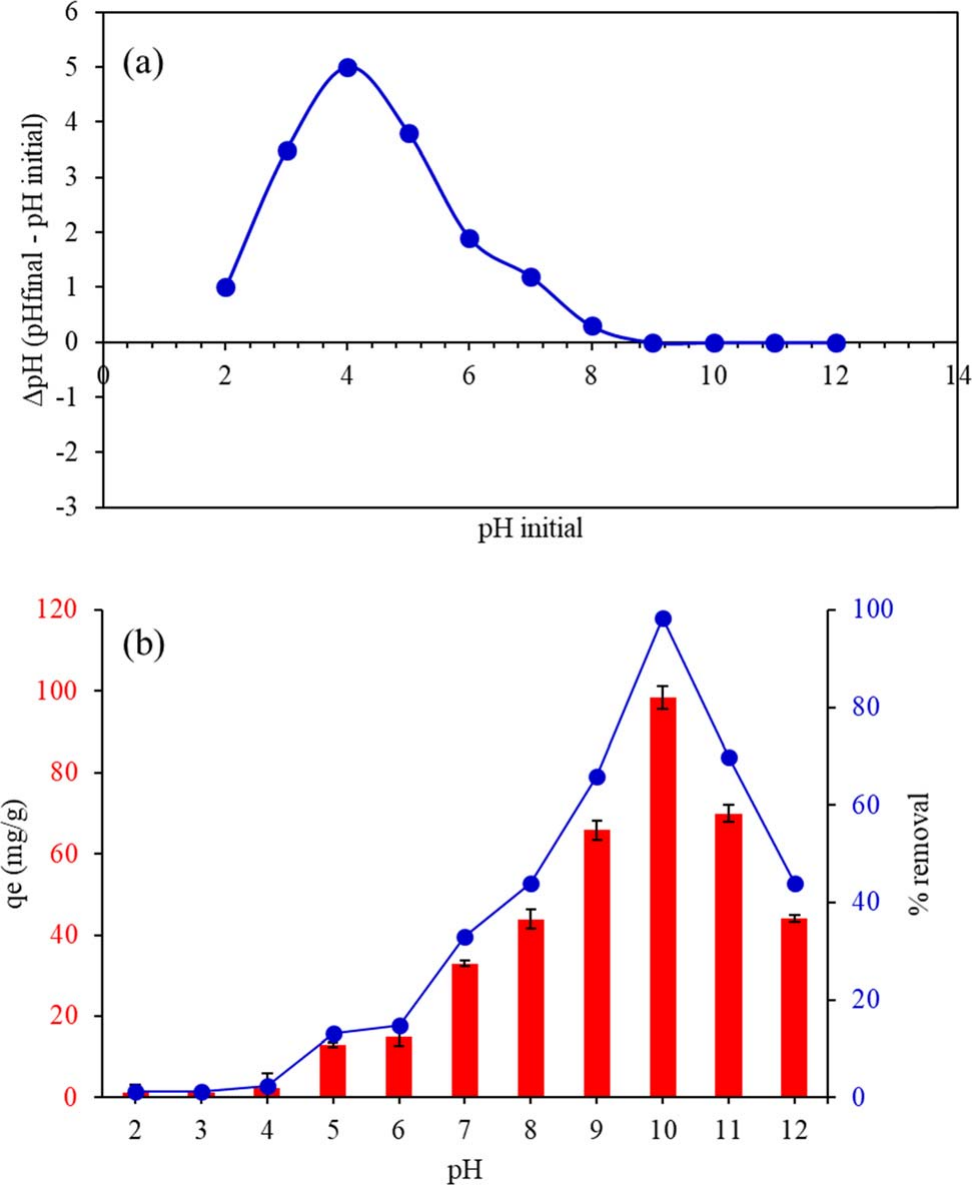
(a) Point of zero charge pH (pH_pzc_) and (b) effect of pH on the adsorption capacity of malachite green by FHAP–GCTS. Conditions: MG concentration, 200 mg L^−1^; sample volume, 10 mL; adsorption temperature, 28 °C; adsorbent dosage, 0.02 g; adsorption time, 10 min.

The pH_pzc_ curve was analyzed to determine the point of zero charge (pH_pzc_) of the material by plotting the difference between the final pH and the initial pH (ΔpH) against the initial pH. Initially, at lower pH values, the ΔpH was positive, indicating that the material had a basic effect, raising the pH of the solution. As the initial pH increased, the ΔpH reached a peak, reflecting the maximum basic effect. Beyond this peak, the ΔpH gradually decreased and eventually crossed the *x*-axis, where ΔpH equaled zero, identifying the pH_pzc_. This point marked the pH at which the material's surface was neutral, with no net charge. Further increases in the initial pH resulted in negative ΔpH values, showing that the material began to have an acidic effect on the solution. The pH_pzc_ was found to be 8.8 for FHAP–GCTS (Fig. 6a). Below pH 8.8, the surface of FHAP–GCTS was positively charged, while above pH 8.8, it became negatively charged. The analysis of the effect of initial pH on the assessment of adsorption efficiency was conducted by performing batch experiments, and the adsorption capacity, *q*_e_ (mg g^−1^), was determined. Experiments were carried out at pH values ranging from 2 to 12. The adsorption capacity of the FHAP–GCTS adsorbent for malachite green is significantly influenced by the pH. Chitosan chains possess large functional groups (–NH_2_, –COOH, –OH) with lone pairs of electrons, leading to changes in surface charges at different pH levels. As depicted in Fig. 6b, at low pH, malachite green is prone to be repelled due to protonation-induced electrostatic repulsion with amino groups of chitosan.^33^ Additionally, in the lower pH range, malachite green competes with H^+^ ions to bind onto the composites. The FHAP–GCTS was found to have limited stability in acidic environments, with its solubility significantly increased as the pH dropped below 7 due to the protonation of its surface sites, which destabilized the material and led to dissolution, especially below pH 5, rendering it unsuitable for applications in acidic conditions.^34^ At pH 10, the surface becomes more negatively charged, creating electrostatic conditions favorable for malachite green. The adsorption of MG onto FHAP–GCTS is high at base pH values. Consequently, pH 10 was utilized for subsequent experiments.

#### 3.7.2. Effect of adsorbent dosage

**Fig. 7:**
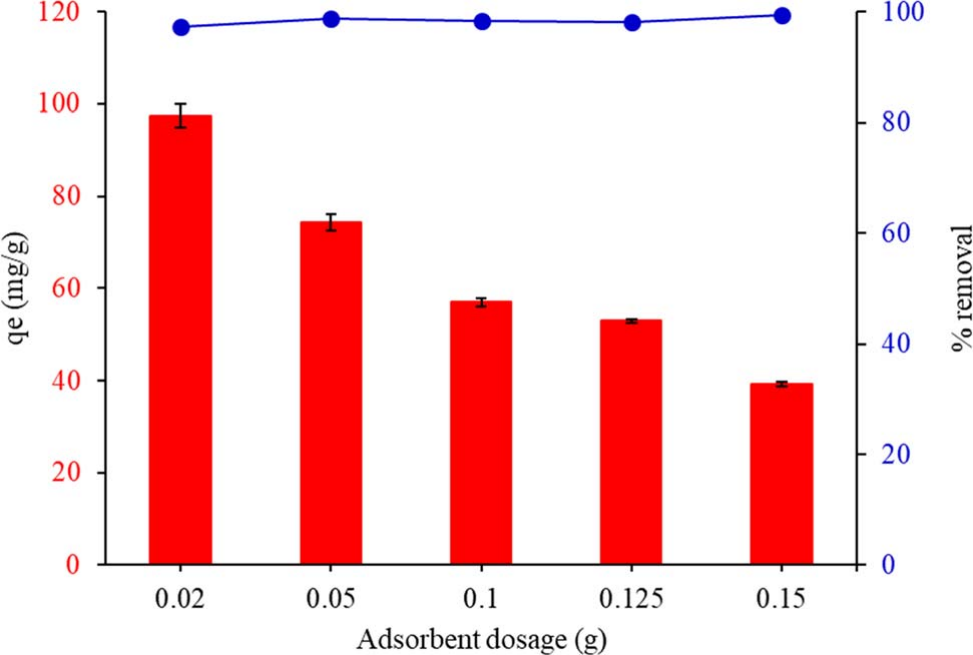
Effect of the adsorbent dosage on the adsorption capacity of malachite green by FHAP–GCTS. Conditions: MG concentration, 200 mg L^−1^; sample volume, 10 mL; pH 10; adsorption temperature, 28 °C; adsorption time, 10 min.

Fig. 7 shows the connection between *q*_e_ and the dosage of the adsorbent. The adsorption capabilities of MG increased rapidly when the adsorbent dosage surpassed 0.02 g. This decrease in the amount of sorbents accessible at the surface for these dyes might be explained by the FHAP–GCTS particles aggregating or overlapping at the adsorption sites. When 0.02 g of FHAP–GCTS powder was used, MG had the greatest *q*_e_. As a result, 0.02 g of adsorbent was used in the trials that followed.

#### 3.7.3. Effect of contact time

**Fig. 8:**
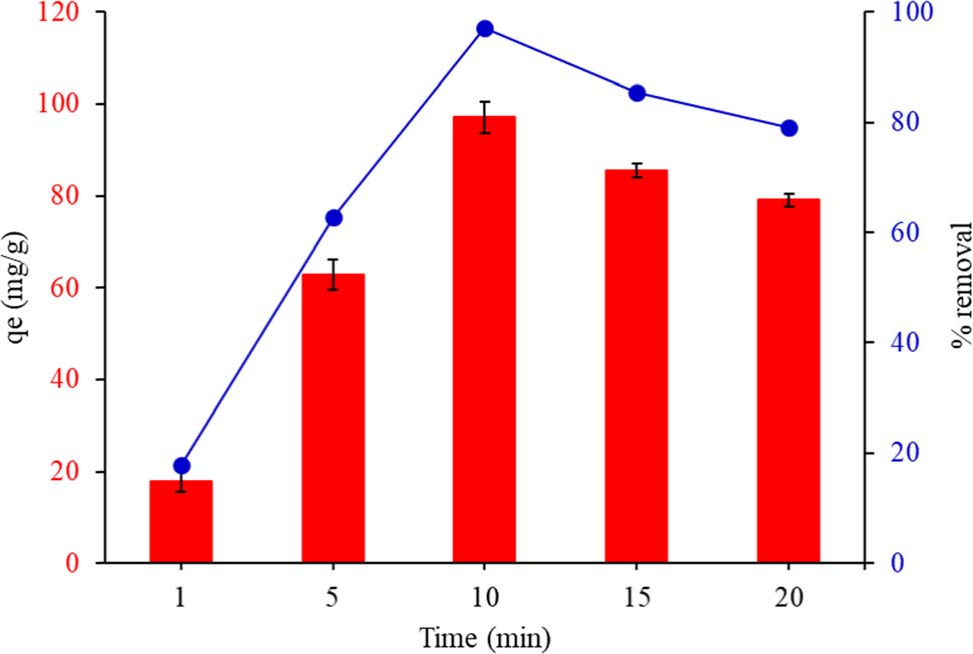
Effect of contact time on adsorption capacity of malachite green by FHAP–GCTS. Conditions: MG concentration, 200 mg L^−1^; sample volume, 10 mL; pH 10; adsorption temperature, 28 °C; adsorbent dosage, 0.02 g.

The adsorption experiments were executed with various contact times (1, 5, 10, 15 and 20 min). Fig. 8 depicts the adsorption capacity and removal% of MG by FHAP–GCTS at different contact times. The rapid transport of the analyte from the aqueous solution to the many active sites on the sorbent's surface is often responsible for the high adsorption rate. It was observed that *q*_e_ and removal% underwent a rapid increase until equilibrium was attained within 10 minutes. When the adsorption time was extended and ultrasonic exposure was increased, heat could be generated within the water medium due to the ultrasonic energy. As a consequence, the temperature of the sample solution could be raised, leading to a decrease in the efficiency of adsorption. To achieve optimal dye adsorption, a sonication time of 10 minutes for MG was chosen in subsequent experiments.

#### 3.7.4. Effect of temperature

**Fig. 9:**
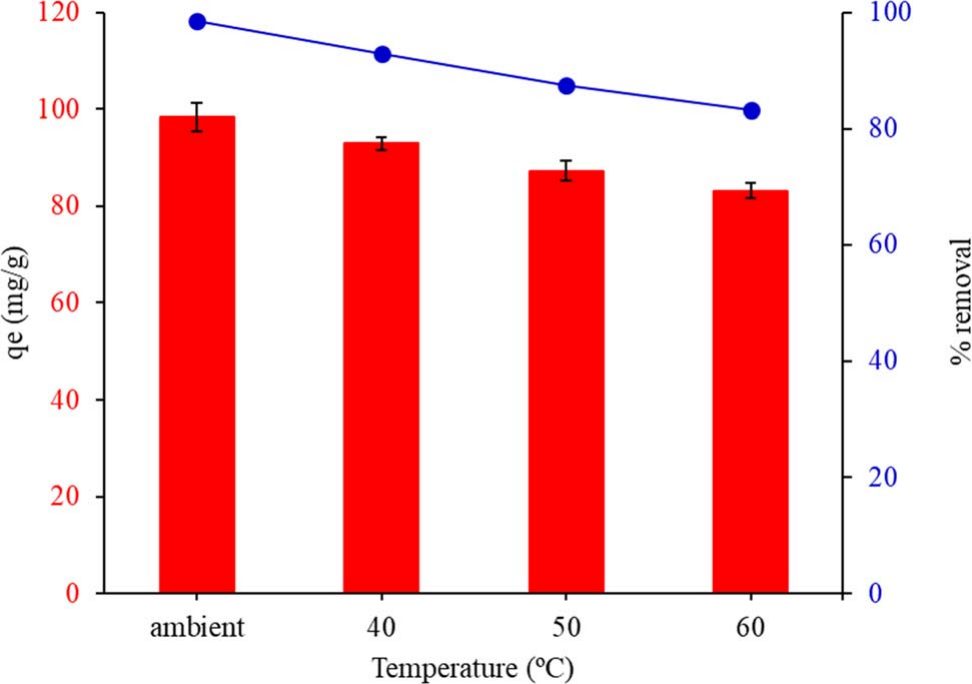
Effect of temperature on the adsorption capacity of malachite green by FHAP–GCTS. Conditions: MG concentration, 200 mg L^−1^; sample volume, 10 mL; pH 10; adsorbent dosage, 0.02 g; adsorption time, 10 min.

Fig. 9 shows the results of an investigation into the impact of temperature on the adsorption capacity and percentage removal of malachite green at various ambient temperatures (28 °C, 40 °C, 50 °C, and 60 °C). The adsorption capacities and removal% decreased as the temperature amplified from 28 °C to 60 °C. The decrease in MG equilibrium adsorption as temperature rises is primarily due to physical adsorption, which is particularly dominant at lower temperatures. For further trials, the ambient temperature was chosen because it showed the most advantageous link between adsorption capacity and adsorbents.

#### 3.7.5. Adsorption mechanism

The uptake of malachite green by FHAP–GCTS was governed by a synergistic mechanism that involved two key interactions: electrostatic interactions and hydrogen bonding. These interactions worked together to optimize the binding of the dye to the sorbent material. Firstly, the electrostatic interactions played a significant role under alkaline conditions. When the pH of the solution exceeded pH_pzc_ of 8.8, the surface of FHAP–GCTS became negatively charged. This negative charge arose due to the deprotonation of functional groups such as hydroxyl and phosphate groups on the hydroxyapatite and amine groups on the chitosan component of the composite. The negatively charged surface then exerted an electrostatic attraction towards the positively charged malachite green dye molecules, which are cationic in nature. This attraction facilitated the initial adsorption of the dye onto the FHAP–GCTS surface, contributing significantly to the overall uptake process. In addition, hydrogen bonds were formed between the malachite green dye molecules and functional groups present on the sorbent surface. Specifically, the hydroxyl groups on hydroxyapatite and the hydroxyl and amine groups on chitosan provided active sites for hydrogen bonding. These groups interacted with the nitrogen and aromatic rings of malachite green, establishing multiple hydrogen bonds. This network of hydrogen bonds stabilized the dye molecules on the surface, reinforcing their attachment and preventing desorption. The combination of these two mechanisms-electrostatic attraction and hydrogen bonding resulted in a highly effective sorption process, making it a highly effective material for the removal of malachite green from water solutions. Consequently, FHAP–GCTS proved to be an efficient sorbent material, capable of addressing pollution caused by malachite green in water treatment applications.

#### 3.7.6. Reusability

After cleaning with a re-extraction solution of ethanol, the FHAP–GCTS can be reused. The reusability of the adsorbent is a significant factor in evaluating the performance of sorption materials. Consequently, the same sorbent underwent three repetitions of sorption–desorption cycles. The operational stability of FHAP–GCTS was maintained throughout the process for three reusable cycles, with no significant loss of sorption capacity (>95%). As a result, the studied MG could be reused at least three times.

#### 3.7.7. Adsorption kinetics

**Fig. 10:**
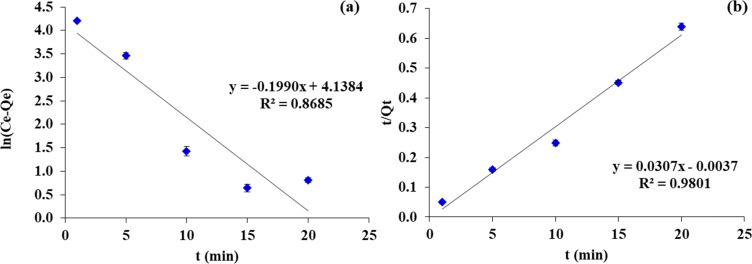
The kinetic data of MG on FHAP–GCTS. (a) Pseudo-first-order kinetic and (b) pseudo-second-order kinetic.

Table 1 provides a summary of the rate determining processes for MG adsorption. Analysis of the linear form of the pseudo-first-order kinetic model indicated its inadequacy for fitting the data (*R*^2^ = 0.8685). The results indicated that pseudo-first-order kinetics were not a governing mechanism for the adsorption of this dye onto FHAP–GTCS. Conversely, the pseudo-second-order kinetic model exhibited *R*^2^ = 0.9801 when fitted to the experimental adsorption data for the dye at equilibrium. This finding suggests that the pseudo-second-order kinetic model provides a more accurate description of MG sorption onto FHAP–GTCS compared to the pseudo first-order kinetic model (Fig. 10).

**Table 1:** MG adsorption kinetics on FHAP–GCTS

*q*_e,exp_ (mg g^−1^)	Pseudo-first order kinetic model			Pseudo-second order kinetic model		
	*q*_e,cal_ (mg g^−1^)	*k*_1_ (h^−1^)	*R* ^2^	*q*_e,cal_ (mg g^−1^)	*k*_2_ (g mg^−1^ h^−1^)	*R* ^2^
40.20	62.70	0.20	0.8685	32.57	0.02	0.9801

#### 3.7.8. Adsorption isotherm

In Table 2 revealed that the Langmuir isotherm model was then employed to calculate a maximum monolayer adsorption capacity of 285.7 mg g^−1^ for MG. Additionally, analysis of the RL value for MG dye indicated that it fell between 0 and 1, suggesting that favorable adsorption of the dye by the adsorbent had occurred. As a result, the adsorption process on the composite FHAP–GCTS was homogeneous and involved a single molecular layer. To assess the adsorption effectiveness of FHAP–GCTS composites.

**Table 2:** Adsorption isotherm for MG on FHAP–GCTS composites

Langmuir model			Freundlich model		
*q*_max_ (mg g^−1^)	*K*_L_	*R* ^2^	1/*n*	*K*_F_	*R* ^2^
285.7	0.00361	0.9851	0.5537	192.753	0.9848

Table 3 presents the adsorption results of various adsorbents for MG. The outcomes reveal the saturation adsorption of MG. These findings collectively suggest that the FHAP–GCTS composites synthesized in this study exhibit favorable adsorption performance for the removal of MG pollutant.

**Table 3:** Comparison of maximum adsorption capacity (*q*_max_) for MG onto FHAP–GCTS with other adsorbents from previous reports

Adsorbent	*q*_max_ (mg g^−1^)	Reference
Almond shell-based biocarbon	166.7	35
Fugus-based biocarbons	176.0	36
Amine-terminated succinic acid-modified magnetic nanoparticles (MSA@TEPA)	282.7	37
Fe–Zn–PVA nanocomposites	92.6	38
Iron modified silica/polyurethane composite (FMS/PU)	34.3	39
Carbon nanospheres (CNSs) derived from oil palm leaves	104.2	40
4AZW zeolite	45.6	41
FHAP–GCTS	285.7	This work

#### 3.7.9. Adsorption thermodynamic

The temperature dependence of adsorption systems and the spontaneity of dye molecule adsorption on FHAP–GCTS were investigated using adsorption thermodynamics. A summary of the calculated values for Δ*H*°, Δ*S*°, and Δ*G*° is provided in Table 4. A negative Δ*H*° confirms the exothermic nature of the adsorption process. The adsorption of MG onto FHAP–GCTS exhibited a heat reaction of −231.36 kJ mol^−1^. The large magnitude of this value also suggests a strong interaction between the dye molecules and the adsorbent, which is consistent with chemisorption. A negative Δ*S*° indicates a decrease in randomness or disorder at the interface during the adsorption process. The value of −368.22 J mol^−1^ K^−1^ suggests that the adsorption process leads to a more ordered system, which is often the case when dye molecules are effectively bound to the adsorbent surface. The negative values of Δ*G*° at all temperatures (301–333 K) suggest that the adsorption between the sorbents and the dye is both feasible and spontaneous. Consequently, the adsorption of MG by the FHAP–GCTS adsorbent is deemed a spontaneous and exothermic process.

**Table 4:** Thermodynamic parameters for MG on FHAP–GCTS composites

Δ*G*° (kJ mol^−1^)				Δ*H*° (kJ mol^−1^)	Δ*S*° (J mol K^−1^)
301 K	313 K	323 K	333K		
−8.49	−4.81	−3.25	−2.46	−231.36	−368.22

## 4. Conclusion

The promotion of sustainability was facilitated by the incorporation of chitosan composite with tilapia fish-scale-derived hydroxyapatite, which involved utilizing a byproduct from the food industry that would otherwise have been considered waste. This approach was aligned with the principles of a circular economy and reduced the environmental impact associated with fish processing. The employment of FHAP–GCTS adhered to sustainable practices and highlighted the ability to transform waste into valuable resources. For modified materials, comprehensive testing and characterization were deemed essential to determine their suitability for specific applications involving the removal of malachite green. Under optimal adsorption conditions (MG concentration, 200 mg L^−1^; sample volume, 10 mL; pH 10; adsorbent dosage, 0.02 g; adsorption temperature, 28 °C; adsorption time, 10 min), the synthesized material exhibited a maximum adsorption capacity of 285.7 mg g^−1^ for MG under optimized conditions. Malachite green was effectively adsorbed by FHAP–GCTS through a combination of electrostatic interactions, which facilitated the binding of the dye to the sorbent surface and significantly contributed to the overall effectiveness of the sorbent in removing the dye from the solution. Hydrogen bonding also played a role, as the hydroxyl groups (–OH) on the surface of hydroxyapatite, along with the hydroxyl and amine groups in chitosan, formed hydrogen bonds with malachite green molecules. The Langmuir isotherm model was effectively employed to describe the adsorption behavior, as evidenced by a high correlation coefficient (*R*^2^ = 0.9851). Thermodynamic analysis indicated that the adsorption process was exothermic, while kinetic studies revealed that the pseudo-second-order kinetic model best represented the adsorption kinetics. Overall, FHAP–GCTS was presented as a promising alternative for the environmentally friendly removal of malachite green from water.

## Data availability

Data are available upon request from the authors.

## Author contributions

Conceptualization, NK, PS, RP, NL, TR, PN, SC and AK; methodology, NK, TR, NS, SL and YT; software, NK, SL and TR; validation, NK, TR, MS and PP; investigation, NK and TR; data curation, NK, PS and NL; writing—original draft preparation, NK and NL; writing—review and editing, NK, PS, SC and NL; supervision, NL, PS, TR and SC; project administration, NL. All authors have read and agreed to the published version of the manuscript.

## Conflicts of interest

The authors declare no competing financial interests.

## Supplementary Material

RA-014-D4RA03102D-s001
