# Supplementary material for: Ultrasound-assisted formation of composite materials from fish scale waste hydroxyapatite in the presence of gamma-irradiated chitosan for the removal of malachite green
Source: RSC Adv. 2024 Oct 1;14(40):29737–47. doi: 10.1039/d4ra03102d (PMC11443413; doi:10.1039/d4ra03102d)
Supplement: RA-014-D4RA03102D-s001 [file RA-014-D4RA03102D-s001.pdf]

Supplementary Information (SI) for RSC Advances.
This journal is © The Royal Society of Chemistry 2024

Supplementary Information

Ultrasound-assisted formation of composite materials from fish scale waste hydroxyapatite in the presence of gamma-irradiated chitosan for the removal of malachite green

Nattaporn Keanjun[1,a], Thitirat Rattanawongwiboon[2,a], Phitchan Sricharoen[3*], Sakchai Laksee[3], Naengnoi Saengsane[4], Yanisa Thepchuay[4], Paweena Porrawatkul[4], Rungnapa Pimsen[4], Arnannit Kuyyogsuy[4], Prawit Nuengmatcha[4], Saksit Chanthai[5], Maliwan Subsadsana[6] and Nunticha Limchoowong[1**]

[1]Department of Chemistry, Faculty of Science, Srinakharinwirot University, Bangkok , 10110, Thailand
[2]Nuclear Technology Research and Development Center, Thailand Institute of Nuclear Technology (Public Organization), Nakhon Nayok 26120, Thailand
[3]Division of Health, Cosmetic and Anti-Aging Technology, Faculty of Science and Technology, Rajamangala University of Technology Phra Nakhon, Bangkok 10800, Thailand
[4]Nanomaterials Chemistry Research Unit, Department of Chemistry, Faculty of Science and Technology, Nakhon Si Thammarat Rajabhat University, Nakhon Si Thammarat 80280, Thailand
[5]Materials Chemistry Research Center, Department of Chemistry and Center of Excellence for Innovation in Chemistry, Faculty of Science, Khon Kaen University, Khon Kaen 40002, Thailand
[6]Program of Chemistry, Faculty of Science and Technology, Nakhon Ratchasima Rajabhat University, Nakhon Ratchasima, 30000, Thailand

Corresponding author; e-mail: phitchan.s@rmutp.ac.th (P. Sricharoen)*
nunticha@g.swu.ac.th (N. Limchoowong)**

[a]These authors contributed equally to this work.

3.2. X-ray diffraction (XRD)

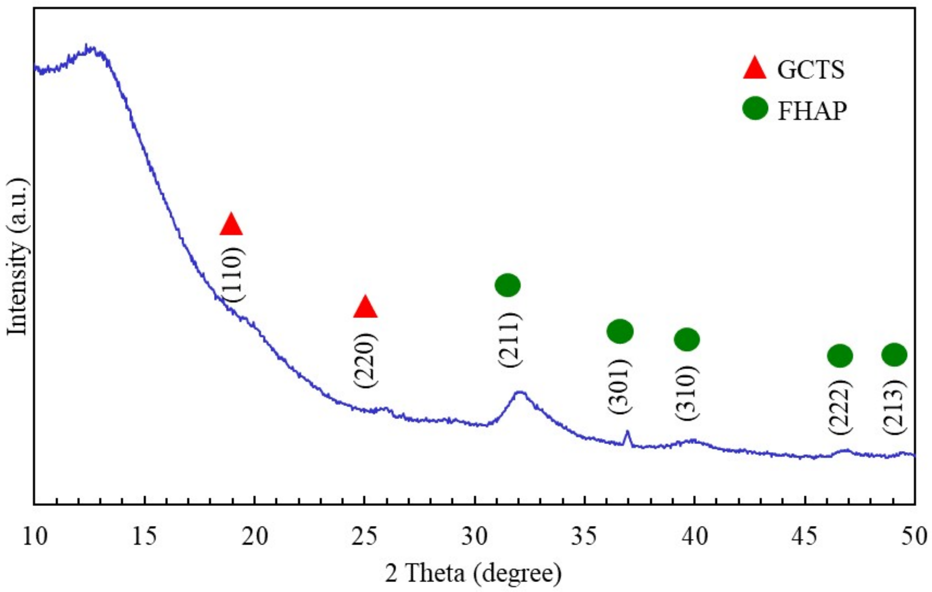

Fig. S1. XRD patterns of FHAP-GCTS composites.
